# ERRATUM: The role of oestrogen receptor α in human thyroid cancer: contributions from coregulatory proteins and the tyrosine kinase receptor HER2

**DOI:** 10.1530/ERC-09-0216e

**Published:** 2024-11-25

**Authors:** Dara O Kavanagh, Marie McIlroy, Eddie Myers, Fiona Bane, Thomas B Crotty, E McDermott, Arnold D Hill, Leonie S Young

**Affiliations:** 1 School of Medicine and Medical Science, UCD Conway Institute, St Vincent’s University Hospital and University College Dublin, Dublin, Ireland; 2 Endocrine Oncology Research Group, Department of Surgery, Royal College of Surgeons in Ireland, St Stephens Green, Dublin, Ireland

The authors and journal apologise for an error in the above paper, which appeared in volume 17 part 1, pages 255-264. The error relates to Fig. 2B given on page 260, in which incorrect immunofluorescent images of breast tumour histology were submitted in error. The corrected figure artwork, including the correct thyroid cancer images, is given below:

**Figure 2:**
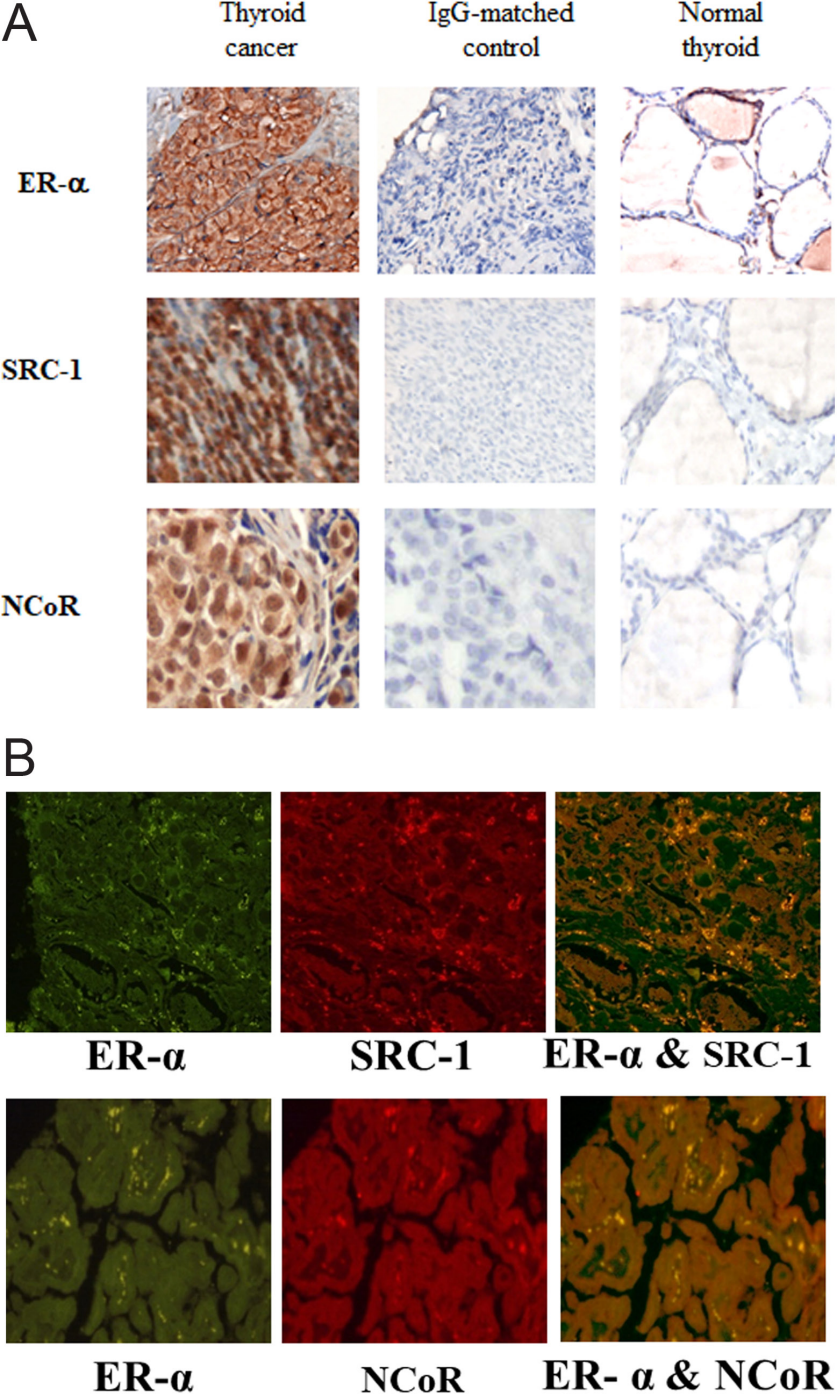
(A) Immunohistochemical localisation of ERa, SRC-1 and NCoR counterstained with haematoxylin and matched IgG-negative controls in human thyroid cancer (200×) and normal thyroid (200×). (B) Immunofluorescent colocalisation of ERa with SRC-1 and NCoR in human thyroid cancer.

